# ATG13 restricts viral replication by induction of type I interferon

**DOI:** 10.1111/jcmm.14483

**Published:** 2019-07-03

**Authors:** Jiang‐long Du, Peng Ma, Chen Wang, Yan Zeng, Yu‐jia Xue, Xue‐cai Yang, Xue‐mei Wan, Fan‐fan Chang, Tian‐yu Zhao, Xiao‐ye Jia, Hai‐zhen Wang, Jing Liu, Zhong‐ren Ma, Xin Cao, Kui‐zheng Cai

**Affiliations:** ^1^ College of Life Science and Engineering, Center for Biomedical Research Northwest Minzu University Lanzhou China; ^2^ State Key Laboratory of Veterinary Etiological Biology Lanzhou Veterinary Research Institute, Chinese Academy of Agricultural Sciences Lanzhou China; ^3^ Hebi Precision Medical Research Institute People's Hospital of Hebi Hebi China; ^4^ Department of Medical Oncology People's Hospital of Hebi Hebi China

Dear Editor,

Autophagy is an intracellular degradation pathway regulated by the orchestrated action of the autophagy‐related (ATG) proteins. ATG proteins traditionally have been studied for their roles in autophagy, but they have increasingly demonstrated functions other than cellular self‐eating.^1^ The non‐autophagic functions of autophagy proteins include cell survival and apoptosis, modulation of cellular traffic, protein secretion, cell signalling, transcription, translation and membrane reorganization.^2^ The ATG12‐ATG5 conjugate negatively modulated immunostimulatory RNA (isRNA)‐generated type I IFN production by sequestration of CARD proteins.^3^ The direct antiviral activity of IFNγ against murine norovirus in macrophages required nondegradative role of ATG5‐ATG12, ATG7 and ATG16L1.^4^ Intriguingly, ATG5‐ATG12 positively regulated antiviral NF‐κB and IRF3 signalling during foot‐and‐mouth disease virus infection.^5^ Moreover, Mauthe et al conduct an unbiased RNA interference screen approach to explore unconventional functions of ATG proteins during viral infections. They revealed that ATG13 and FIP200 act independently of the ULK complex to modify picornaviral replication outside the context of autophagy.^6^, ^7^, ^8^ Previous study suggest that ATG13 had both autophagic and nonautophagic functions and that the latter were essential for cardiac development.^9^ Targeting the ATG101‐ATG13 interaction showed the strongest autophagy‐inhibitory effect.^10^ However, the role of ATG13 in innate antiviral immune signalling remains to be elucidated.

In order to identify possible roles for ATG13 in antiviral immunity, we transfected human embryonic kidney cells HEK293T (293T cells) with an IFN‐sensitive response element (ISRE) luciferase reporter, which was an interferon regulatory factor 3 (IRF3)‐dependent promoter, and the internal control renilla luciferase, as well as expression vectors containing ATG13. Then we treated the cells intracellularly with the synthetic nucleic acid duplex poly(I:C)/ poly(dA:dT) or infected with vesicular stomatitis virus (VSV) for 12 hours to trigger type I interferon signalling. The ISRE‐Luc activity induced by intracellular poly(I:C)/ poly(dA:dT) or VSV infection was greatly enhanced by HA‐ATG13 overexpression (Figure 1A). These results indicated that ATG13 may be a positive regulator of the type I interferon response. Next, we co‐transfected ATG13 with signalling proteins involved in innate antiviral response and determined the activation of ISRE or nuclear factor kappa B (NF‐κB) promoters. The overexpression of ATG13 with the retinoic acid‐inducible gene‐I (RIG‐I)‐like receptors (RLRs) signalling proteins, including RIG‐I, melanoma differentiation‐associated antigen 5 (MDA5), mitochondrial antiviral signalling (MAVS), tumor necrosis factor (TNF) receptor associated factor (TRAF) family member associated NF‐κB activator (TANK) binding kinase 1 (TBK1), inhibitor of NF‐κB kinase subunit epsilon (IKKi), IRF3 and stimulator of interferon genes, additively activated ISRE luciferase activity (Figure 1B). However, overexpression of ATG13 with RIG‐I and MDA5, but not TRAF6 and MAVS, additively activated NF‐κB luciferase activity (Figure 1C). Intriguingly, ATG13 itself can activate ISRE and NF‐κB luciferase activity in a dose‐dependent manner (Figure 1D). To demonstrate a link between the enhanced type I interferon response and antiviral immunity of ATG13 gene, we overexpressed ATG13 in 293T cells and then infected the cells with VSV tagged with enhanced green fluorescent protein (VSV‐eGFP) at a multiplicity of infection of 0.1. Overexpression of ATG13 rendered the cells resistant to viral infection and resulted in considerably fewer GFP^+^ (virus‐infected) cells than among cells transfected with empty vector (Figure 1E). Besides, we performed RT‐qPCR to quantify VSV replication over a time course manner (Figure S1).

**Figure 1:**
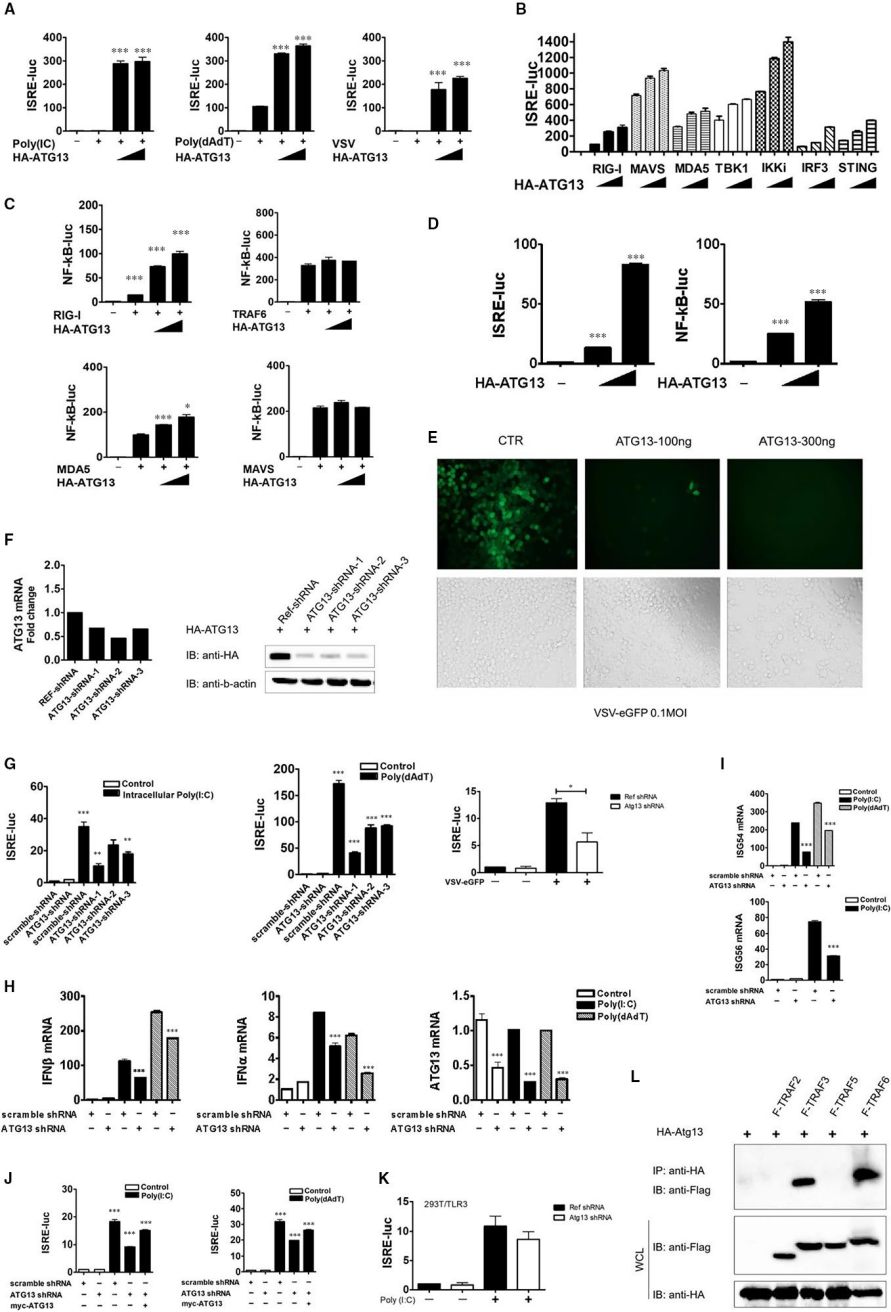
ATG13 is a positive regulator of type I interferon signalling. (A), 293T cells (2 × 10^5^) were plated in 24‐well plates and transfected, through the use of Lipofectamine 2000, with plasmid encoding an IFN‐sensitive response element (ISRE) luciferase reporter (firefly luciferase; 100 ng) and pRL‐TK (renilla luciferase plasmid; 10 ng) together with increasing concentrations (0, 100 or 200 ng) of plasmid encoding HA‐ATG13, followed by no treatment or treatment with intracellular poly(I:C) (2 μg/mL), poly(dA:dT) (1 μg/mL) or VSV (MOI, 0.01). Reporter gene activity was determined by normalization of the firefly luciferase activity to renilla luciferase activity. The comparisons were performed by student's *t* test between the indicated bars and the positive control, which are treated by intracellular poly(I:C), poly(dA:dT) or VSV without HA‐Atg13. (B), 293T cells in 24‐well plates were transfected with plasmid encoding an ISRE luciferase reporter (firefly luciferase; 100 ng) and pRL‐TK (renilla luciferase plasmid; 10 ng) together with 100 ng plasmid encoding Flag‐tagged RIG‐I, MDA5, MAVS, TBK1, IKKi, IRF3 or STING and increasing concentrations (0, 100 or 200 ng) of plasmid encoding HA‐ATG13. Empty pcDNA3.1 vector was used to maintain equal amounts of DNA among wells. Cells were collected at 24 h after transfection and luciferase activity was measured. (C), 293T cells in 24‐well plates were transfected with plasmid encoding an NF‐κB luciferase reporter (firefly luciferase; 100 ng) and pRL‐TK (renilla luciferase plasmid; 10 ng) together with 100 ng plasmid encoding Flag‐tagged RIG‐I, MDA5, MAVS or TRAF6 and increasing concentrations (0, 100 or 200 ng) of plasmid encoding HA‐ATG13. Luciferase activity was measured similar to B. (D), ISRE and NF‐κB luciferase activities were measured as described in A‐C, without any ligand or signal molecules stimulation. (E), 293T cells in 24‐well plates were transfected with empty vector or different dose of HA‐ATG13, followed by infection of VSV‐eGFP at an MOI of 0.1. Cells were observed under phase‐contrast and fluorescence microscope. Original magnification, ×10. (F), Knockdown efficiency of three ATG13 shRNA was detected by RT‐qPCR and Immunoblot analysis. For immunoblot, 293T cells were transfected with plasmids encoding scramble or ATG13 shRNA for 48 h, then transfected HA‐ATG13 for 24 h. Exogenous protein level of ATG13 was detected by anti‐HA. (G), 293T cells (1×10^5^) were plated in 24‐well plates and transfected with plasmids encoding scramble or ATG13 shRNA for 48 h, then transfected luciferase reporter plasmid for 12 h, followed by treatment with intracellular poly(I:C) (2 μg/mL), poly(dA:dT) (1 μg/mL) or vesicular stomatitis virus (VSV) (MOI, 0.01) for 12 h. ISRE and NF‐κB luciferase activities were measured as described above. (H), 293T cells (1 × 10^5^) were plated in 24‐well plates and transfected with plasmids encoding scramble or ATG13 shRNA for 60 h, followed by treatment with intracellular poly(I:C) (2 μg/mL) or poly(dA:dT) (1 μg/mL) for 12 h. IFN‐β, IFN‐α and ATG13 transcripts were detected by RT‐qPCR. (I), 293T cells were treated the same as described in H. ISG54 and ISG56 transcripts were detected by RT‐qPCR. (J), 293T cells (1 × 10^5^) were plated in 24‐well plates and transfected with plasmids encoding scramble or ATG13 shRNA for 48 h, then transfected luciferase reporter plasmid and myc‐ATG13 for 12 h, followed by treatment with intracellular poly(I:C) (2 μg/mL) or poly(dA:dT) (1 μg/mL) for 12 h. ISRE luciferase activities were measured as described above. (K), 293T‐TLR3 stable cells were transfected with plasmids encoding scramble or ATG13 shRNA for 48 h, then transfected luciferase reporter plasmid for 12 h, followed by treatment with poly(I:C) (10 μg/mL) in the culture medium for 12 h. ISRE luciferase activities were measured as described above. (L), HA‐ATG13 and FLAG tagged TRAF2, TRAF3, TRAF5 or TRAF6 were co‐transfected in 293T cells. Co‐IP assay was performed 24 h after transfection

Then we determined whether specific knockdown of endogenous ATG13 would decrease IFN‐β expression under physiological conditions. We used three ATG13‐specific short hairpin RNA (shRNA) constructs to knock down the expression of ATG13. All three efficiently inhibited the expression of endogenous ATG13 in mRNA level and transfected ATG13 in protein level in 293T cells (Figure 1F). With the ISRE luciferase reporter assay, we found that knockdown of ATG13 resulted in much less activity of the ISRE luciferase reporter triggered by intracellular poly(I:C), poly(dA:dT) or VSV‐eGFP in 293T cells (Figure 1G). Consistent with that, knockdown of ATG13 also resulted in lower expression of IFN‐β and IFN‐α mRNA with intracellular poly(I:C) or poly(dA:dT) treatment (Figure 1H). Several interferon‐stimulated genes (ISG), including ISG54 and ISG56, were down‐regulated by ATG13 knockdown with the same treatment (Figure 1I). For rescue experiment, ISRE luciferase activity recovered to some extent with ATG13 re‐expression after knockdown under intracellular poly(I:C) or poly(dA:dT) treatment (Figure 1J). For 293T‐TLR3 cells (293T cells that express TLR3) treated with poly(I:C), no significant difference was observed in ISRE activity with ATG13 knockdown (Figure 1K). It suggest that ATG13 may not get involved in TLR3 signalling pathway. Finally, we identified that ATG13 could interact with TRAF3 and TRAF6 by co‐immunoprecipitation assay (Figure 1L). It's well documented that TRAF3 and TRAF6 are key adaptors in ISRE and NF‐κB signalling pathway respectively.

Altogether, our results suggest that ATG13 may regulate viral replication by potentiation type I interferon signalling. TARF3 and TRAF6 may be the important target of ATG13 to regulated type I interferon production. Further studies are needed to investigate the detailed mechanisms of ATG13 in the regulation of type I interferon.

## CONFLICT OF INTEREST

The authors confirm that there is no conflict of interest.

## Data Availability

The data that support the findings of this study are available from the corresponding author upon reasonable request.
